# Increasing use of systems science in cardiovascular disease prevention to understand how to address geographic health disparities in communities with a disproportionate burden of risk

**DOI:** 10.3389/fcvm.2023.1216436

**Published:** 2023-07-13

**Authors:** Kyla L. Bauer, Krista A. Haapanen, Nathaniel Demeke, Meredith P. Fort, Kamal H. Henderson

**Affiliations:** ^1^ Department of Health Systems, Management and Policy, Colorado School of Public Health, University of Colorado Anschutz Medical Campus, Aurora, CO, United States; ^2^ Department of Human and Organizational Development, Vanderbilt University, Nashville, TN, United States; ^3^ Department of Cardiology, University of Colorado Denver School of Medicine, Aurora, CO, United States

**Keywords:** health status disparities, health equity, systems analysis, systems theory, cardiovascular diseases/prevention & control, social determinants of health

## Abstract

**Objective:**

Marginalized communities shoulder a disproportionate burden of cardiovascular disease (CVD) driven by concentrated neighborhood social risk factors. We provide a case study of systems science application to address geographic CVD health disparities at the community level – informing the science of CVD health disparities research.

**Methods:**

We conducted a two-phased, multi-methods needs assessment in the Denver, Colorado area. Phase I consisted of a social network analysis to map a two-mode network of existing CVD prevention interventions and their implementing organizations. In Phase II, group model building (GMB) sessions with key community, public health, and healthcare provider stakeholders, were utilized to identify and visualize community factors contributing to disparities in CVD risk, producing a consensus-based causal loop diagram.

**Results:**

Between May 2021 and June 2022, we conducted 24 virtual, semi-structured interviews in Phase I to describe CVD prevention interventions, and 7 virtual GMB sessions in Phase II to describe experiences of disparities in CVD risk. For the purposes of this paper, we focus on a subset of results for both phases. In Phase I we identified 89 active CVD prevention interventions, 29 of which addressed tobacco use. In Phase II, causal loop diagrams revealed root causes of disparities in CVD risk. We provide an example of a causal loop diagram that focuses on the community prevalence of tobacco use, identifying stress as a key underlying factor driving disparities. The integration of findings from both phases highlighted the alignment and misalignment between quit tobacco intervention goals and how they are being experienced in marginalized communities.

**Conclusion:**

Systems science methods were useful to organize a large number of CVD prevention efforts, and evaluate the root causes of CVD health disparities in a high risk community. By integrating these two aspects, interventions may be reoriented to more effectively address the root causes of CVD health disparities.

## Introduction

Healthy People 2030 establishes U.S. national public health goals that prioritize achieving health equity, which requires eliminating health disparities arising from social determinants of health and health literacy (1). Health disparities, or differences in health linked with social, economic, and environmental disadvantage, are well known to exist in CVD risk factors and outcomes (2–5). In 2015 the American Heart Association (AHA) issued a scientific statement highlighting “the most significant opportunities for reducing death and disability from CVD in the United States lie with addressing the social determinants of cardiovascular outcomes” (4).

The factors known to increase CVD risk are wide-ranging, from socioeconomic to psychosocial factors (4, 5). In combination, these factors make it possible to predict geographic areas of communities at highest CVD risk (5). While social determinants that drive disparities in CVD outcomes have been identified in research, strategies for addressing them are, to date, underdeveloped.

One primary strategy is to prioritize policy, systems and environmental (PSE) approaches to reduce disparities. For example, the Centers for Disease Control and Prevention has recommended action, such as regulating tobacco marketing, rather than solely relying on individual tobacco cessation interventions to reduce disparities in tobacco use (6). Similarly, the Institute of Medicine has identified PSE approaches to reduce disparities in population hypertension, emphasizing public health partnership interventions that improve density of supermarkets and safe physical activity spaces in inner cities (7).

When PSE approaches regarding CVD disparities are viewed through a health equity lens, however, it becomes evident that such a strategy is highly complex. Any health disparity is the end result of a complex and dynamic system occurring at the population level (8). Complex adaptive systems consist of different actors responding in dynamic ways with one another, creating feedback loops and emerging patterns of interactions between socio-ecological levels (8). Actors—e.g., policies, public health funding, local environment, etc. – all work in unique, often non-linear, and evolving ways to influence CVD disparities. Disentangling how best to address this complex system for more effective PSE approaches requires a modern methodological perspective.

Systems science methods were developed to help model complex and dynamic systems to identify key relationships, that when modified will have the greatest impact on improving population outcomes and reduce the likelihood of unintended consequences (9). The first step in systems science methods is building understanding around the system model (9). Studies on healthy eating, weight management, physical activity and tobacco use have constructed systems models to understand relationships among influential factors in environments and communities, to tease out which relationships to focus on to improve population health outcomes (10–13).

Incorporating health disparities into these models has identified additional factors that help explain ways in which PSE changes can better reach marginalized populations, avoiding unintended consequences (11, 14, 15). A comprehensive look at CVD prevention interventions, within communities of higher CVD risk and the context of health disparities in which they exist, has not yet been done to guide policy and population health leaders to identify key changes that can reduce geographic disparities in CVD risk.

Here we discuss the Cardiovascular Disease Equitable Prevention in Communities (CVD-EPIC) study. It is an example of how to use systems science to understand health disparities in CVD prevention, to make it more accessible to CVD prevention researchers. This community case study presents an overview of the two-phases of CVD-EPIC that aimed to:

1) Understand the network of actors involved in implementing CVD prevention interventions in Denver and their collaborative efforts;
2) Develop visual diagrams that capture the factors related to disparities in CVD risk faced by marginalized populations in Denver, Colorado and adjacent communities.

Our goal in describing the approach and initial findings from this project is to share an example of how to use systems science to understand health disparities in CVD prevention, that makes it more accessible to CVD prevention researchers and practitioners. Future research intends to use this knowledge, to develop a CVD prevention strategy in Denver that addresses health disparities in CVD risk.

## Context

This study was approved as exempt from human subject research by the University of Colorado Institutional Review Board with IRB #21-3314. It is a multi-method, community-based participatory study conducted within a local public health jurisdiction. Its aim is to describe CVD interventions within the context of geographic health disparities. It took place in two phases, from May 2021 to June 2022, in the area of Denver, Colorado, USA.

In the United States, public health programs are primarily delivered through the support of local governments (16). Denver is a unique local government jurisdiction that is simultaneously a city and a county. The study was primarily focused on communities within the Denver area, but did spill over into adjacent communities within Aurora, Colorado, as well. The Institute for Public Health at Denver Health, a local health-focused agency within the largest safety net hospital in Denver, and Denver Department of Public Health and Environment, the local public health department, have historically shared local government responsibility for public health initiatives in Denver (17). They were identified as key partners and consulted early on to shape development of the research study.

Denver City and County was found to have high CVD risk at the county level, based on a prior social risk index of counties within the state of Colorado that incorporated percent of racial-ethnic minorities, college educated residents, and poverty (K. Henderson, personal communication, June 16, 2021). Study participants in both phases either played a key role in CVD prevention activities in Denver area communities, worked directly with high CVD risk sub-populations and/or were a member of a high CVD risk sub-population. Further participant recruitment details are provided later on.

## Frameworks

To help classify CVD prevention primary goals and interventions, we used a combination of two frameworks. Life's Simple 7® is a list of evidence-based, individual activities promoted by the American Heart Association, that are known to reduce CVD risk (18). The same, or a similar set of activities, are commonly used in nationwide CVD health initiatives (2). At the time our study began, we did not include “Get Healthy Sleep” because Life's Essential 8^TM^ had not yet been published (19). Activities focused on cardiovascular risk factors in our study included tobacco use, healthy weight, nutritious diet, physical activity, and managing diabetes, cholesterol and blood pressure. Since Life's Simple 7® goals can be achieved through a variety of mechanisms, it was necessary to incorporate an additional framework to ensure a wide variety of CVD prevention interventions were considered. The Prevention Impacts Simulation Model (PRISM) incorporates both individual-level and population-level interventions for CVD prevention, that are either prescriptive or facilitative, described by four broad categories: clinical, behavioral support, taxes and regulation, health promotion and access (20). PRISM is a system dynamics model that is capable of comparing the future impact of changing more than one CVD prevention intervention on population CVD risk through simulations. The benefit of using PRISM as a framework is that it is based on a credible systems model with adaptability to specific socioeconomic factors of different geographic areas (20).

## Phase I: existing network of CVD prevention interventions

Social network analysis has been used in public health to examine the social ecological context prior to implementing a specific program, or a broad public health activity, to improve its efficacy (21–23). When addressing health equity as a part of CVD prevention in Denver area communities, key stakeholders conveyed that many diverse CVD prevention programs already existed, but there was no comprehensive list that explained what was already being done. This created a major gap in understanding how to approach health equity without knowing the current system already in place. Defining the system of CVD prevention from the perspective of those involved, was broken down into two components: (1) identify active CVD interventions being implemented in the Denver area, that targeted at least one of Life's Simple 7® activities; (2) understand the organizational context of these CVD interventions. A two-mode network approach was used to describe relationships among CVD interventions and the organizational network in place to implement or deliver them.

## Participants

In this phase, semi-structured interviews in the style of a guided survey were conducted on a total of 24 participants from public health, clinical, state and local government, and community organizations (Table 1). A purposive and snowball sampling approach was initiated, with key informants identified as leaders in chronic disease prevention in the Denver area. These key informants were asked to identify activities and organizations they thought were vital to their organization's local work preventing CVD. Organizations they named were then contacted, introduced to the study, and invited to identify additional activities and organizations, using the same criteria. We conducted interviews until CVD interventions from all four PRISM categories had been included.

**Table 1:** Study participants from Phases I and II.

Phase I	No.	Phase II*	No.
Local Government	5	Government and Public Administration	2
State Government	1	Public Health Service	4
Health System	9	Healthcare Service	3
Clinical Community Center	2	Health Prevention Community Organization	2
Pharmacy	2	Community Member	5
Health Prevention National Non-Profit	4		
Health Prevention Community Organization	1		
Total	24		14

* Two participants self-identified as working in two industries.

## Network analysis methods with preliminary results

From April to October of 2021, we conducted semi-structured interviews (Supplementary Table S1). Interviews lasted up to 45 min and were transcribed. Participants were asked to name all of the CVD interventions with which they directly worked. Interventions were organized by a PRISM model category, and which of Life's Simple 7® activities were targeted. When interventions could not be categorized within the frameworks provided, a new category was created. Participants also listed up to eight primary collaborators, defined as anyone working outside their immediate department, division, or organization, who helped with implementing each intervention, and the strength of their relationship. If there were more than eight collaborators, participants provided an estimate of total collaborators to understand the breadth of collaboration. If time allowed, follow-up questions were asked about specific CVD interventions to glean in-depth knowledge. Usually there was only sufficient time to ask follow-up questions for one or two of the CVD interventions listed.

**Figure 1:**
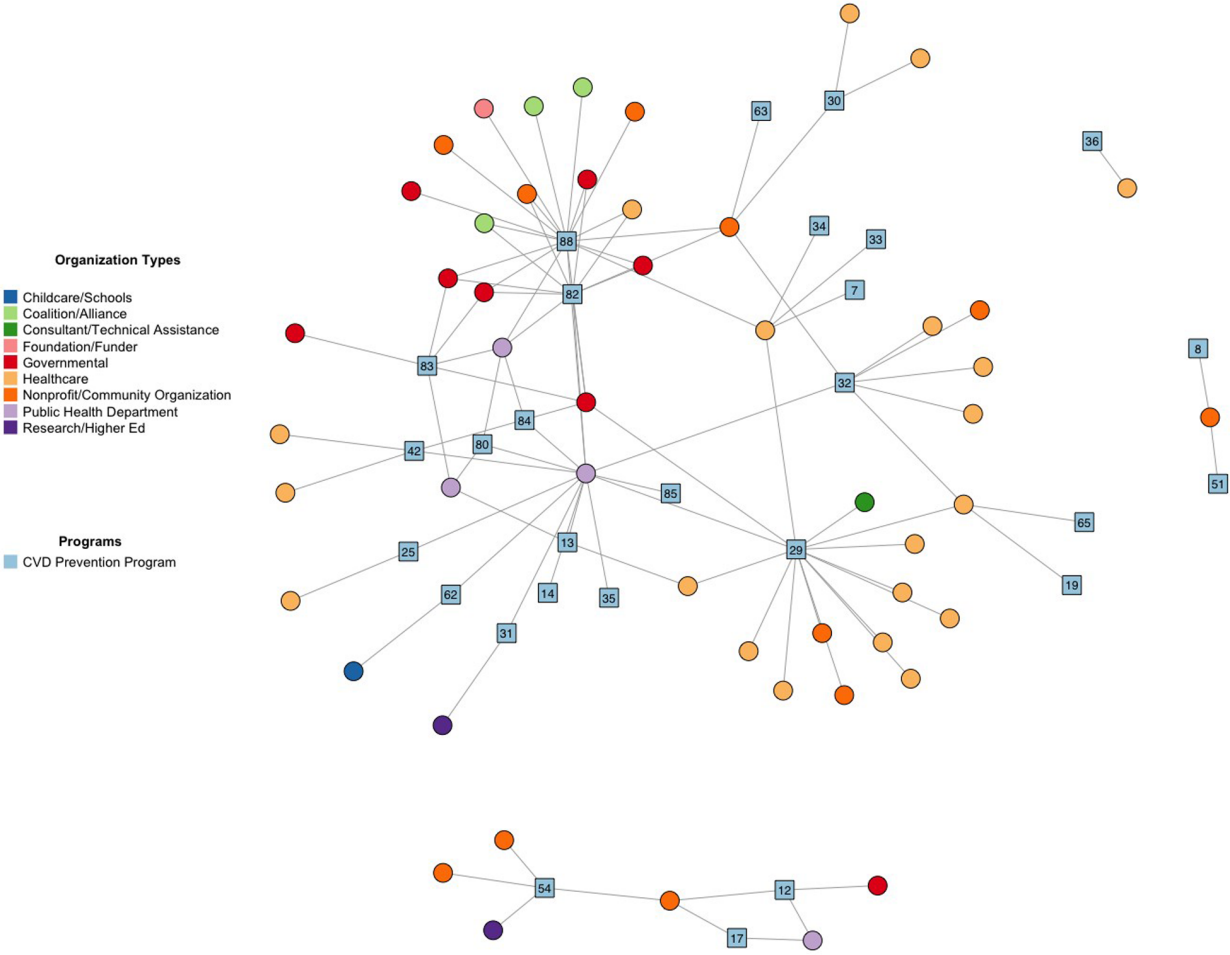
Social network plot of key partnerships implementing “Quit Tobacco” prevention activities in denver area. “Quit Tobacco” prevention programs are blue squares. Implementing organizations are circles. Participants were allowed to identify up to 8 key partnerships represented by undirected lines. Coalitions represent a large group of organizations up to 100+.

We identified a total of 89 active CVD interventions in the Denver area. To give an example of results, we will focus on the 29 active interventions that specifically worked towards the goal of “Quit Tobacco” from Life's Simple 7® (Table 2). Of the 29 interventions, 38% were classified as clinical, 38% as behavioral support, 31% as health promotion and access, and 21% as policy (taxes and regulation), though several overlapped in more than one PRISM category. Network analysis demonstrated which “Quit Tobacco” interventions had the greatest quantity of collaboration, and which types of organizations were more often collaborating with each other (Figure 1).

**Table 2:** CVD prevention activies focused on “Quit Tobacco” goal in denver area.

#	Program/activity Name	Clinical	Behavior support	Health promotion	Policy
7	Tobacco Cessation Program		X		
8	Beyond Hunger Nutrition Program		X		
12	Aging Mastery		X	X	
13	Media Campaign for Tobacco Cessation		X	X	
14	Colorado Heart Healthy Solutions		X	X	
17	Journey to Wellness		X	X	
19	Adult Wellness Program		X	X	
25	Ambulatory Care Services	X			
29	E-Referral to Colorado Quitline	X			
30	Get with the Guidelines	X			
31	Healthy Hearts	X			
32	Target BP	X			
33	Clinical Prevention (Primary Care)	X			
34	Clinical Rehab for CVD	X			
35	Screening for CVD Risk Factors	X			
36	Pre-screening at Health Fairs	X			
42	Healthy Behavior Screening Tools	X	X		
51	General Fitness Program		X		
54	Check. Change. Control.	X	X	X	
62	Vaping Reduction Education			X	
63	Workplace Health Achievement Index			X	
65	School-based Heart Health Education Program			X	
80	Smoking Complaint Program				X
82	T21 Licensing Program				X
83	Underage Tobacco Sales Compliance Program				X
84	Colorado Clean Indoor Air Act				X
85	Denver Smoke Free Multi-Unit Housing				X
88	Tobacco Flavor Ban		X		X

Note: Clinical: Individual-level prescriptive interventions; Behavioral Support: Individual-level facilitative interventions; Health Promotion & Access: Population-level facilitative interventions; Policy (Taxes & Regulation): Population-level prescriptive interventions.

E-Referral to the Colorado Quitline (#29) was emphasized in interviews as a key clinical resource that was heavily promoted through media campaigns, and had multiple healthcare and community non-profit organizations working with the program (Table 2 and Figure 1). The Tobacco Flavor Ban (88) was a new policy proposal that several coalitions, health and community non-profits, and government agencies were working on to draft and pass. Coalitions were often large groups, ranging from dozens to over a hundred collaborating organizations, that were commonly involved in working on new policy proposals. Existing legislation, such as the Underage Tobacco Sales Compliance Program (83) or the Colorado Clean Indoor Air Act (84), primarily involved only public health departments and government agencies for implementation. Several behavioral and clinical interventions had only one to a few organizations working on them.

## Phase II: health disparities related to CVD risk factors

The goal of Phase II was to understand the complex social and environmental context for health disparities, that affect CVD risk and outcomes across communities in the Denver area. Group model building is a participatory systems science method that visualizes community context through the visual aid of causal loop diagrams (12, 13, 24–26). The process of group model building is iterative, working with a variety of stakeholders to help articulate a problem and build shared understanding around it (24–26). Emphasis is placed on shared understanding from experience, rather than requiring evidence-based knowledge. However, it is possible to incorporate or compare causal loop diagram results with evidence-based knowledge, through initial or follow-up literature reviews. The causal loop diagram is the end product, made up of interconnected factors that influence each other and lead to a primary outcome, creating an overall picture of a problem's complexity (10–12, 27). Group model building was used in this phase, in order to develop causal loop diagrams that capture how health disparities related to CVD are experienced in the Denver area by marginalized populations.

## Participants

A total of 14 participants from public health, clinical, or community groups were involved in group model building sessions (Table 1). Some participants from Phase I were asked to participate in Phase II. Purposive and snowball sampling were used to identify additional stakeholders. All community members had personal experience or interest in CVD prevention in their communities, but it was not a requirement to participate. Group model building sessions continued until thematic saturation had been reached, meaning no new additional factors contributing to health disparities were identified, and responses had become repetitive.

## Group model building and causal loop diagram methods with preliminary results

From April to June of 2022, we conducted 7 virtual group model building sessions with 2 to 3 participants. Sessions lasted for up to 90 min. Variables for causal loop diagrams were captured for four of the Life's Simple 7® risk factors: not smoking, healthy eating, physical activity, and healthy weight. These were selected because they are lifestyle behaviors that generally promote health, to reduce risk of CVD without necessarily having developed CVD nor requiring clinical care (28). We intend to continue future studies that incorporate the clinical behaviors of managing blood pressure, controlling cholesterol, and reducing blood sugar.

Before a participant was scheduled to attend a virtual session, they were sent a short video introducing the main concepts behind causal loop diagrams to help set expectations. A publicly-shared script from Scriptapedia, for causal mapping with seed structure, was modified to guide the structure of the group model building sessions (29). At each session, the research team would provide at minimum a modeler and a facilitator. When possible, a recorder was also present to take notes, but all sessions were virtually recorded for future transcription. The modeler drew causal loop diagrams in real time, based on group feedback using the online application Miro Online Whiteboard (no version provided). Our modeler was a principal investigator who had the most experience with group model building. The facilitator presented and supported discussion around the main problem of health disparities related to CVD risk. Presentation of the problem was standardized, by using a script developed by the research team, to easily explain important concepts around health disparities and the goal of the research study (Supplementary Script S1).

Groups were provided with an example of a seed model, and a list of evidence-based factors from literature review, that contribute to CVD health disparities for each of the four CVD prevention goals (Supplementary Figure S1). The facilitator emphasized that the seed model and factors were merely prompts to consider, but groups were encouraged to generate their own ideas based on experience, and even disagree with or leave out the prompts. When groups listed immediate factors contributing to disparities, they were also encouraged to consider upstream factors, to generate relationships among factors represented by arrows. Follow-up questions and verifying accurate translation of participants' responses further supported discussion.

**Figure 2:**
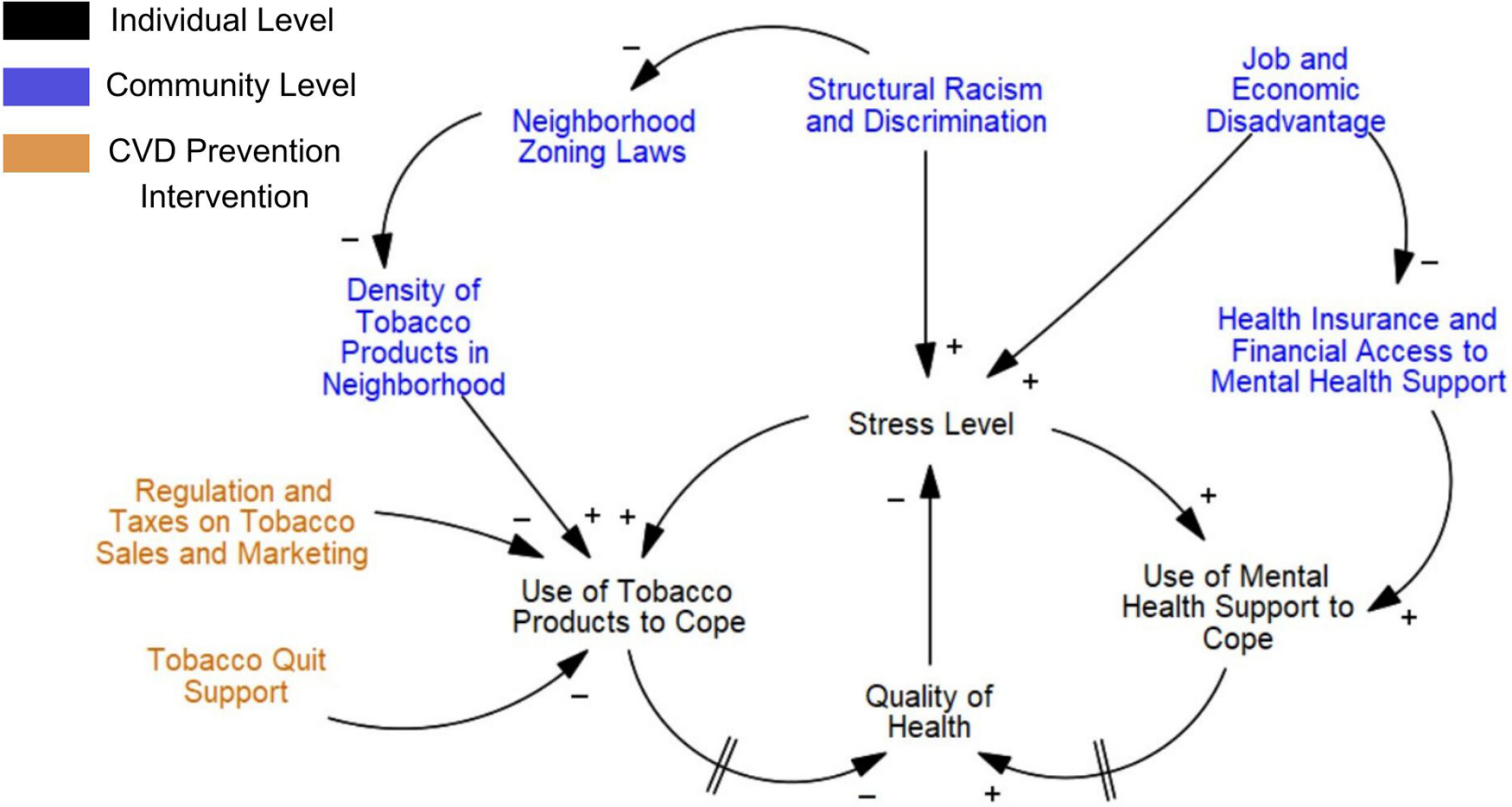
Causal loop diagram example on health disparities in tobacco use in the Denver area, focused on stress. This causal loop diagram was developed based on the experiences of participants. The goal is to understand why there is a geographic disparity in tobacco use prevalence between communities. Differences in factors contributing to stress, and then subsequent differences in access to tobacco products and mental health services to reduce stress levels, create a disparity in tobacco use prevalence. Arrows indicate the direction of relationships between factors. A positive symbol signifies a positive correlation. A negative symbol signifies an inverse correlation. Two dashes on an arrow reflect a delay in outcome.

Using “Quit Tobacco” as an example of how causal loop diagrams were built, the research team combined the seven group models constructed during sessions into one diagram. First, a comprehensive list of all factors contributing to disparities in tobacco use prevalence in the Denver area was created, with immediate factors contributing to tobacco use prevalence organized into a left-hand column, and upstream factors organized into right-hand columns. Our research team found the easiest starting point was to build several seed models that were able to focus on a specific disparity contributing to differences in tobacco use prevalence, such as stress and differential access to stress coping mechanisms (Figure 2). In this way, causal loop diagrams could incorporate sufficient detail to reflect participants' experiences and distinguish between disparities showing up in different socio-ecological levels (e.g., individual-level disparities vs. community-level disparities). Future work will experiment with building a unified causal loop diagram for each CVD prevention risk factor, and compare them to evidence-based knowledge.

## Combining Phase I and Phase II preliminary results

Combining qualitative, network and group model building analysis produced a rich understanding of how the current CVD prevention system within a geographic area is being experienced differently, and potential sources of modification to reduce disparities identified by the community. For example, in Phase II group model building, participants frequently brought up stress and general mental well-being as a key part of understanding tobacco addiction (Figure 2). One community member stated, “The issue is the community doesn’t want to talk about smoking, they want to talk about mental health, and mental health is directly related to smoking.” Another community member discussed that therapists did not seem trained to address tobacco addiction treatment as a part of stress management, a gap in mental health and medical services he wanted to bridge.

“Quit Tobacco” interventions identified in Phase I qualitative and network analysis did not mention stress as an intervention focus nor disparities in mental health access. While many “Quit Tobacco” interventions were implemented at a population level and were considered a form of policy, the focus of interventions were primarily on reducing access to tobacco products. A difference in neighborhood zoning laws that can restrict retail tobacco sales, was addressed in both research phases as a key disparity current tobacco interventions are considering. However, current interventions are not coupled with ways to increase access to other forms of stress relief within communities, such as mental health access, for communities facing high levels of overall stress. By using multiple systems analysis approaches, it became evident when public health efforts aligned with community need, or when they fundamentally lacked overlap.

The combination of qualitative information from network analysis and group model building demonstrated that those working closely with community members while implementing CVD interventions were often aware of root causes for health disparities identified by community members. However, they did not always feel empowered to address root causes. One expert expressed frustration when trying to reverse geographic trends in tobacco retail density that was related to greater infractions in tobacco youth sales, “To be able to go back and get those zoning laws changed and get those proximity restrictions added after the fact when things are already there, it’s an even harder battle uphill.” Instead, there was a greater focus on policy to improve enforcement of restricting underage tobacco sales.

Similarly, there was recognition that tobacco cessation programs were trying to improve outreach to marginalized communities by increasing diversity in campaigns, ads and staffing, but this did not address the core distrust in healthcare institutions and programs from historical and current racism. As one expert commented, “There may be a little more effectiveness if these programs are designed inherently in the community and driven by them, not just faces on ads or screens, and I’m not saying that we don’t have enough minorities working in these spaces, but I’m talking about the overall design.”

## Discussion

When addressing CVD prevention to improve population health, there is a growing emphasis on policy, systems, and environmental (PSE) approaches to be more effective, and better reach marginalized populations left out of clinical or individual-level interventions (3, 4, 6, 7). It is important to recognize, though, that PSE approaches are not inherently designed to fix population health disparities, unless making those disparities a focal point. Our community case study demonstrated that, within a geographic area classified as having high CVD risk, there were 29 active “Quit Tobacco” interventions out of a total of 89 active CVD prevention interventions. Of the “Quit Tobacco” interventions, 52% were identified as being implemented at the population level, with 21% specifically focused on policy interventions.

Regardless of population- or individual-level approaches, there was tension from both leaders in CVD prevention and community members that tobacco use interventions were insufficient to address root causes for tobacco addiction. Addressing disparities as a key component of this study illuminated additional considerations in tobacco use prevention, such as disparities in neighborhood zoning laws predicting tobacco product prevalence, the stress burden associated with racism and economic disadvantage, and disparities in accessing mental health services.

Based on the group model building sessions, participants talked about stress as being an underlying factor contributing to tobacco addiction that should be recognized within programs and activities. Several strategies were identified for shifting activities to address stress as a part of disparities in tobacco addiction. These included: increasing access to mental health services, and bridging training with mental health providers related to quit tobacco strategies. Colorado has recently launched a statewide mental wellness program, for youth to access a limited number of free counseling sessions, called I Matter, as well as a crisis hot line for suicide and substance use disorder interventions at any age (30, 31). It demonstrates potential policy changes that could be expanded to replace the Colorado Quitline, a call line solely focused on quit tobacco strategies, that has a high degree of collaboration among leaders in CVD prevention and is heavily promoted in Denver. Instead, call lines focusing on offering free or reduced-cost mental health interventions could be more effective and still allow for quit tobacco strategies to be incorporated.

Systems studies on chronic disease prevention have found that when focusing on health disparities in a prevention system, new key factors are often uncovered to explain the health disparity that had not previously been considered in prevention efforts (11, 14, 15). When considering health equity in tobacco control, a causal loop diagram built from literature review found similar root causes contributing to disparities as our study, such as structural racism, low-wage work, and tobacco retailer density (11). This suggests changing the focus of CVD interventions to root causes driving tobacco use disparities, such as low-wage work. In contrast, without a systems approach, a more common way suggested to achieve health equity in tobacco studies, is to tailor interventions to high-risk populations. This may include mobile cessation programs that are more accessible for low-income populations, or catering cessation language to high-risk demographics (32–34). Either a systems or non-systems approach to health equity may be effective, but it is important to recognize that they solve different problems.

It is worth further exploration to better understand why root causes for CVD-risk disparities are not showing up in the overall goals and design of CVD prevention research and interventions. Our study used the Life's Simple 7® framework, a common list of lifestyle and clinical goals to promote evidence-based practices that reduce CVD risk in individuals (18, 35, 36). Limiting the type of CVD interventions included in our Phase I to evidence-based practices ended up mostly excluding interventions that would address root cause disparities in tobacco use. PRISM, the second framework we used in our study, does acknowledge that racism or socio-economic status, for example, are known to have significant impacts on CVD population health outcomes, but these factors are currently used solely to understand differences in impact of CVD interventions, rather than be built into the model itself for intervention (20). This underscores the important influence of framework choice on guiding CVD prevention research and interventions.

Once we integrated more than one systems science method in our study, root causes for CVD risk disparities were discussed, and the full scale of the disparities were better understood. Systems science methods are still relatively new to prevention science research, and using more than one systems science method is even less common (37, 38). After using multiple systems science methods, community health studies on obesity prevention captured a more thorough understanding of community context and helped identify new progress measures to improve intervention efficacy (37, 39). Even outside of health research, multi-method approaches to systems science has been generally found to improve research contributions to solving complex problems (40).

A key goal of the initial phases of our CVD-EPIC study was to develop a shared understanding around health disparities in CVD prevention in a specific geographic location. The results presented in this paper are a first step in considering how to transition current CVD prevention efforts to better address root causes of health disparities in CVD risk. These findings will inform local public health experts and community members engaged in CVD prevention work.

## Limitations

There are several limitations to consider. While this was a multi-methods two-phase study, it was not exhaustive and it is possible that there are additional CVD interventions that were not identified, or CVD prevention organizations that were overlooked. Public health delivery can vary widely across the U.S. based on several factors such as population size, governance, and financing (41). The Denver area is within a specific state and local public health jurisdiction, in a metropolitan area that may have different capacity for public health delivery compared to rural counties or counties within other states, that may impact results.

We found several benefits to using systems science methods to have a more comprehensive understanding of CVD prevention at the community level, however, analysis is time intensive. The time to process and analyze data was considerably longer than the data collection itself, underscoring the need for collaborative CVD prevention research teams and appropriate funding even at a pilot stage. Ideally, we had hoped to get feedback from group model building participants to improve internal validity of causal loop diagrams, for example, but we ran out of time. Instead, we will rely on evidence-based knowledge for comparison.

## Conclusion

Multiple systems science methods were useful to better understand geographic disparities in CVD risk across communities in the Denver area. A large number of interventions at the population and individual level to reduce CVD risk were found to already exist within the Denver area. The approach of this community case study improves understanding of the root causes of disparities in CVD risk that may reorient current CVD interventions to better address health disparities.

## Data Availability

Anonymized data that support the findings of this study are available from the corresponding author, KLB, upon reasonable request.
