# Supplementary material for: Increasing use of systems science in cardiovascular disease prevention to understand how to address geographic health disparities in communities with a disproportionate burden of risk
Source: Front Cardiovasc Med. 2023 Jul 13;10:1216436. doi: 10.3389/fcvm.2023.1216436 (PMC10374219; doi:10.3389/fcvm.2023.1216436)
Supplement: Supplementary file 1 [file Table1.docx]

**Supplementary Table 1. Key Informant Guided Social Network Survey**

| **Resource Generator** | **Choices** |
| --- | --- |
| Could you please list all of the CVD prevention activities or programs that you currently or recently implemented, or that you have other involvement with? | Open-Ended |
| How would you categorize [resource]? | Clinical, Behavioral Support, Health Promotion and Access, Policy and Regulation |
| **Name Generator** |  |
| Could you please list each of your collaborators for [resource]? | Open-Ended |
| How closely did you work with [collaborator]? | Fully Integrated, Partnership, Collaboration, Coordination, Cooperation, Communication, Not Integrated |
| How often did you work with [collaborator]? | Open-Ended |
| **Follow-Up Questions** |  |
| What is the main goal of [resource]? | Open-Ended |
| Are there criteria for participating in [resource]? | Yes, No |
| How is [resource] primarily funded? | Insurance, Grant, Government Tax, Out-of-Pocket, Other |
| How do participants pay to participate in [resource]? | Insurance, Out-of-Pocket, No Charge, Other |
| Was [resource] designed by your organization or was it developed elsewhere? | Designed by Organization, Adopted From Elsewhere, Other |
| Does [resource] have a protocol or guideline to implement it? | Yes, No |
| Has [resource] gone through a formal evaluation? | Yes, No |
| How well has [resource] done at achieving its primary goal? | Poor, Fair, Moderate, Good, Excellent, Other |
| How would you rate the importance of [resource] to CVD prevention in Denver? | Extremely Important, Very Important, Moderately Important, Slightly Important, Not At All Important |
| To what extent does [resource] aim to reduce health disparities in CVD prevention? | Not At All, Somewhat, A Good Amount |
| How often does your department/unit offer [resource] to participants? | Daily, Weekly, Monthly, Annually, Infrequently |
