# Supplementary material for: Increasing use of systems science in cardiovascular disease prevention to understand how to address geographic health disparities in communities with a disproportionate burden of risk
Source: Front Cardiovasc Med. 2023 Jul 13;10:1216436. doi: 10.3389/fcvm.2023.1216436 (PMC10374219; doi:10.3389/fcvm.2023.1216436)
Supplement: Supplementary file 2 [file Table2.docx]

**Supplementary Script 1. Lead Into Group Model Building Discussion**

“Hi [name]. Welcome to our research study. Thank you so much for taking the time to meet with us today. [Quick intro of researchers]. Just to be sure, are you okay with meeting for up to 1 ½ hours today?

I’m going to do a quick review of what we’re working on and our goals for today. [CONSENT: Since this is the first time we are interviewing you, we want to make sure that you fully understand what this research project is about and what it means to participate in it. It’s important that you feel confident and comfortable about participating]. Then I’m going to start interviewing you. [Research partner] is here as well and they will be assisting us with drawing out the information you give us and also asking questions. Did you get a chance to watch the video we sent?

**STEP 1**

**IF NO**: Okay, not a problem. Our research is about understanding why there are major differences in cardiovascular health in Denver. What Dr. Kamal Henderson has already found out is that low-income neighborhoods and minority populations, specifically African-American and Latino populations in Denver, are disproportionately affected by cardiovascular disease. What we want to understand is based on your [job expertise / experience], what do you think might be the most important factors that are contributing to these large disparities in cardiovascular health in Denver. We are interviewing a number of people in different jobs who work in preventing and treating cardiovascular disease in Denver, as well as community members with personal experience navigating this issue. In the end, we are going to combine all of the pictures we draw based on these unique insights, into one comprehensive picture of the most important factors causing disparities in cardiovascular health in Denver. Do you have any questions based on what I said?

**IF YES:** Okay, so just a quick recap – we are going to focus on asking you based on your [job expertise / personal experience] working in [preventing / treating] cardiovascular disease in Denver, what do you think are the most important factors that are contributing to large disparities in cardiovascular health for specific populations in Denver? Dr. Kamal Henderson has found that low-income neighborhoods and minority populations, specifically African-American and Latino populations, in Denver are the most affected by disparities in cardiovascular health. We are going to draw the insight you have into a picture of the most important factors that are causing these differences. Do you have any questions based on what I said or something that you watching in the video?

**STEP 2**

**IF ALREADY INTERVIEWED:** Okay, moving on. Next I would like to ask you some simple questions about your job. In our last interview we already went over some of these questions, so I’m just going to ask for your confirmation. [Confirm]

**IF NEW INTERVIEW:** Okay, now I’m going to share what is called a consent form to go over what it means to participate in this research project and make sure you feel comfortable moving forward with participating. [CONSENT]

Okay, now that you have consented, I’m going to start the interview by asking you some simple questions about your job. [Ask job-related questions]

**STEP 3**

Okay, we are moving on to asking you about your experience in [ prevention / treatment / personal] of cardiovascular health. This is the part where we are going to start drawing. If you look at the board that is up on the screen, you can see 7 boxes that include 7 behaviors that are well known in research to have a big impact on the cardiovascular health of individuals. [Name off 7 factors]. What we want to understand based on your expertise, is what could be driving disparities in these behaviors for Denver residents living in low-income neighborhoods and African-American and Latino populations?

To give you an example, we are going to start by focusing on the prevention of smoking in the first place and then cessation for those already using tobacco products. We have a general idea based on research that there can be a difference in people’s ability to access tobacco cessation programs. So we wrote that down on a post-it note as being an important factor that is causing this outcome. But we could continue to build on that idea and say that the reason some people aren’t able to access tobacco cessation programs is because they don’t know they exist in the first place. So a difference in knowledge about these programs is affecting access to the programs.

If you’re struggling to come up with ideas, we have general knowledge about why these things may be happening in Denver based on research literature, but sometimes this general understanding doesn’t necessarily apply to Denver or to these specific populations. So we might provide suggestions for ideas, but that doesn’t necessarily mean they are right and you can disagree. Along those same lines, [research partner] is going to try to translate what you are saying into a post-it note. If you find that what we write down in the post-it note is not exactly correct, then please feel free to stop us and correct us.

Do you have any questions before we get started?

[Specific prompts for quitting tobacco, maintaining a healthy weight, achieving recommended physical activity levels, and maintaining a healthy diet].

To get you started, we will share an example from research related to smoking and tobacco use. We know that one of the factors affecting tobacco use is **ability to access tobacco cessation programs**. So we wrote that down on a post-it note on the Miro Board as being an important factor that is causing this outcome. But we could also continue to build on that idea and say that the reason some people aren’t able to access tobacco cessation programs is because they don’t know they exist in the first place. So a **difference in knowledge** about these programs is affecting **access to the programs**, which in turn leads to disparities in tobacco use and cardiovascular disease.”
