# Supplementary figures and images for: Increasing use of systems science in cardiovascular disease prevention to understand how to address geographic health disparities in communities with a disproportionate burden of risk
Source: Front Cardiovasc Med. 2023 Jul 13;10:1216436. doi: 10.3389/fcvm.2023.1216436 (PMC10374219; doi:10.3389/fcvm.2023.1216436)

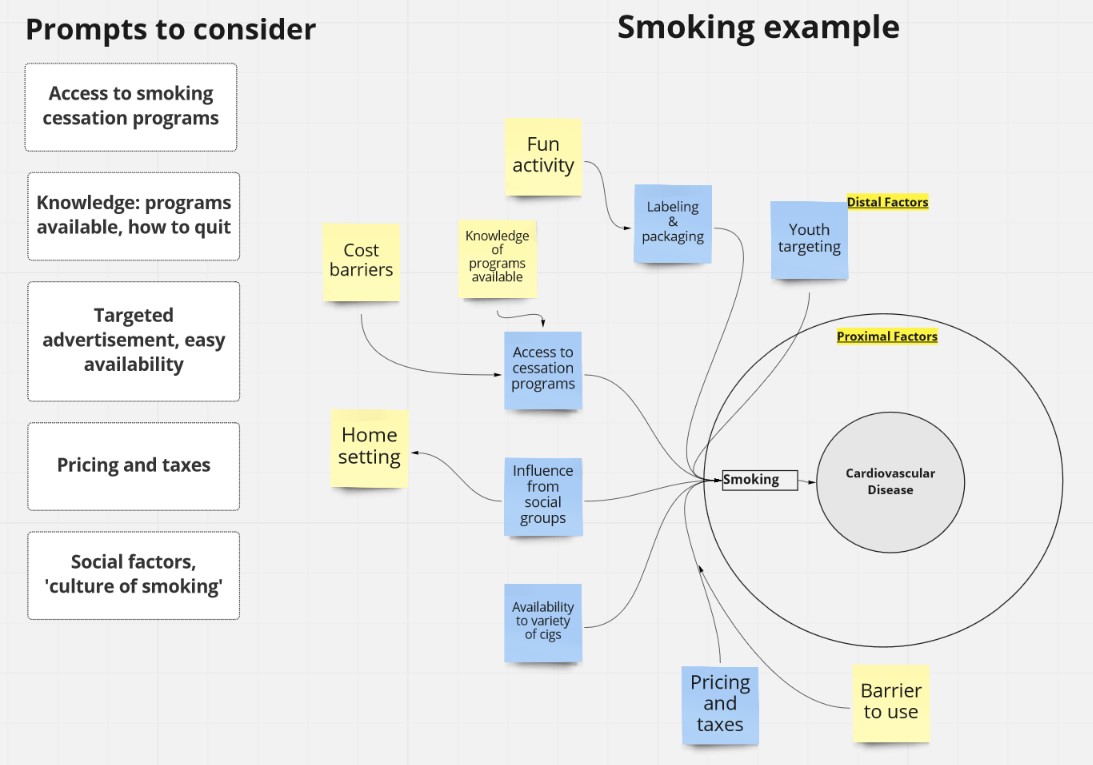

Supplement: Supplementary file 3 [file Image1.jpeg]
